# The OVAREX study: Establishment of ex vivo ovarian cancer models to validate innovative therapies and to identify predictive biomarkers

**DOI:** 10.1186/s12885-024-12429-w

**Published:** 2024-06-07

**Authors:** Lucie Thorel, Jordane Divoux, Justine Lequesne, Guillaume Babin, Pierre-Marie Morice, Romane Florent, Guillaume Desmartin, Lucie Lecouflet, Chloé Marde Alagama, Alexandra Leconte, Bénédicte Clarisse, Mélanie Briand, Roman Rouzier, Léopold Gaichies, Sandrine Martin-Françoise, Jean-François Le Brun, Christophe Denoyelle, Nicolas Vigneron, Corinne Jeanne, Cécile Blanc-Fournier, Raphaël Leman, Dominique Vaur, Martin Figeac, Matthieu Meryet-Figuiere, Florence Joly, Louis-Bastien Weiswald, Laurent Poulain, Enora Dolivet

**Affiliations:** 1 INSERM U1086 ANTICIPE (Interdisciplinary Research Unit for Cancers Prevention and Treatment), BioTICLA Laboratory (Precision Medicine for Ovarian Cancers), Université de Caen Normandie, Caen, France; 2 Comprehensive Cancer Center François Baclesse, UNICANCER, Caen, France; 3 ORGAPRED Core Facility, US PLATON, Université de Caen Normandie, Caen, France; 4 Clinical Research Department, Comprehensive Cancer Center François Baclesse, UNICANCER, Caen, France; 5 Department of Surgery, Comprehensive Cancer Center François Baclesse, UNICANCER, Caen, France; 6 Biological Resource Center ‘OvaRessources’, US PLATON, Université de Caen Normandie, Caen, France; 7 Calvados General Tumor Registry, Comprehensive Cancer Center François Baclesse, UNICANCER, Caen, France; 8 Department of Pathology, Comprehensive Cancer Center François Baclesse, UNICANCER, Caen, France; 9 Department of Cancer Biology and Genetics, U1245 “Cancer and Brain Genomics”, Comprehensive Cancer Center François Baclesse, UNICANCER, Caen, France; 10 US 41 - UAR 2014 - PLBS, University of Lille, CNRS, Inserm, CHU Lille, Institut Pasteur de Lille, Lille, France

**Keywords:** Ovarian cancer, Patient-derived tumor organoids, Patient-derived tumor xenografts, Explants, Spheroids, Predictive functional assays.

## Abstract

**Background:**

Ovarian cancer is the first cause of death from gynecological malignancies mainly due to development of chemoresistance. Despite the emergence of PARP inhibitors, which have revolutionized the therapeutic management of some of these ovarian cancers, the 5-year overall survival rate remains around 45%. Therefore, it is crucial to develop new therapeutic strategies, to identify predictive biomarkers and to predict the response to treatments. In this context, functional assays based on patient-derived tumor models could constitute helpful and relevant tools for identifying efficient therapies or to guide clinical decision making.

**Method:**

The OVAREX study is a single-center non-interventional study which aims at investigating the feasibility of establishing in vivo and ex vivo models and testing ex vivo models to predict clinical response of ovarian cancer patients. Patient-Derived Xenografts (PDX) will be established from tumor fragments engrafted subcutaneously into immunocompromised mice. Explants will be generated by slicing tumor tissues and Ascites-Derived Spheroids (ADS) will be isolated following filtration of ascites. Patient-derived tumor organoids (PDTO) will be established after dissociation of tumor tissues or ADS, cell embedding into extracellular matrix and culture in specific medium. Molecular and histological characterizations will be performed to compare tumor of origin and paired models. Response of ex vivo tumor-derived models to conventional chemotherapy and PARP inhibitors will be assessed and compared to results of companion diagnostic test and/or to the patient’s response to evaluate their predictive value.

**Discussion:**

This clinical study aims at generating PDX and ex vivo models (PDTO, ADS, and explants) from tumors or ascites of ovarian cancer patients who will undergo surgical procedure or paracentesis. We aim at demonstrating the predictive value of ex vivo models for their potential use in routine clinical practice as part of precision medicine, as well as establishing a collection of relevant ovarian cancer models that will be useful for the evaluation of future innovative therapies.

**Trial registration:**

The clinical trial has been validated by local research ethic committee on January 25th 2019 and registered at ClinicalTrials.gov with the identifier NCT03831230 on January 28th 2019, last amendment v4 accepted on July 18, 2023.

## Background

### Ovarian cancer: epidemiology and therapeutic management

Ovarian cancers are responsible for over 207.000 deaths worldwide in 2022, and in 80% of epithelial ovarian carcinoma cases the diagnosis is made at an advanced stage (FIGO III/IV), making it the first cause of death from gynecological malignancies [1, 2]. Optimal surgery and platinum-based chemotherapy are the basis of the treatment of epithelial ovarian cancers. The treatment timeline will be based on the stage, resectability of the carcinomatosis, histological type and comorbidities of the patients. Even if first-line carboplatine/paclitaxel combination achieves response rates close to 80%, among patients whose tumors were initially sensitive to treatment, 75% relapse within 18 months, eventually developing chemoresistance [3]. The introduction of new treatments and the evolution of protocols over the last thirty years have only marginally improved overall survival, which remains around 45% at 5 years [4]. In ovarian cancers, innovative treatments are struggling to become established, and the only recognized and used prognostic factors (i.e. impacting management modalities) are stage of dissemination, residual tumor mass after excision, histology and the homologous recombination (RH) status. The development of new therapeutic strategies likely to overcome chemoresistance therefore remains a major challenge.

Over the years, targeted therapies such as antiangiogenic treatments and PARP inhibitors (PARPi) have been developed first as a treatment for recurrences before being recommended in first line, thanks to their effectiveness. Anti-angiogenic therapies (bevacizumab) have found their place in the management of these cancers with a real benefit in terms of quality of life, but very modest in terms of overall survival [5, 6]. However, it still showed greater effectiveness in at-risk groups (inoperable stage III, unable to be debulked to < 1 cm maximum disease, and stage IV disease) [5, 7]. In the other hand, PARPi have revolutionized the therapeutic management of epithelial ovarian cancers (EOC) [8]. All the different trials showed a significant improvement of progression-free survival in patients with EOC, in first-line and second-line or later maintenance therapy. However, PARPi provided the greatest clinical benefit in patient tumor carrying BRCA1/BRCA2 mutation or exhibiting homologous recombination deficiency (HRD). Indeed, PARP enzymes play a role in DNA repair and their inhibition leads to an accumulation of single and then double-strand breaks that will cause synthetic lethality in an HRD context. Although there is no companion test for carboplatin or bevacizumab, some have been developed for PARPi such as Myriad test or GIScar based on the HRD signature [9, 10]. The development of a companion test is a key step in the development of new therapies to enable personalized medicine: having a suitable treatment for presumed sensitive tumors and avoiding unnecessary and potentially toxic treatment for patients. Functional tests could therefore be used to improve HR status profiling and accurately identify HRD tumors, as well as enabling the implementation of companion tests for other treatments [11].

### Predictive functional assays

Functional precision medicine is a strategy whereby live tumor cells from patients are directly exposed to drugs to provide translatable, personalized information to guide therapy [12]. This approach generates dynamic, functional data that may highlight key vulnerabilities not necessarily driven by genomic alterations. Predictive functional assays rely on the ex vivo (or in vivo) modelling of a patient tumor from pathologically-qualified samples obtained during a medical procedure such as diagnosis biopsy, primary tumor or metastasis resection, blood containing circulating tumor, ascites, etc… Tumor samples are generally processed to primary cultures retaining the original features of the tumor cells of the patient and exposed to treatments of interest. This allows to determine their functional profile (sensitivity/resistance to treatment, ability to repair DNA, mitochondrial apoptotic priming, etc…) using different methods (viability/cytotoxicity assays, real-time imaging, histology/immunohistochemistry, BH3 profiling…). This profile can be used afterwards for predictive purposes and thus guide clinical decision making [12]. Such predictive functional assay can be performed on various biological materials and tumor models as detailed thereafter.

### Tumor models

Developing functional precision medicine requires advanced experimental models to properly predict the behavior of a complex system such as cancer. In the past decades, much progress has been made in developing representative cancer models using in vitro, ex vivo and in vivo approaches that mirror cancer pathogenesis, tumor heterogeneity and angiogenesis [13]. Among others, they include ex vivo models such as patient-derived tumor organoids (PDTO) [14], spheroids from ascites [15] and tissue slices [16–18] or in vivo models such as patient-derived xenograft (PDX) models [19].

PDX models are established by transplanting human tumors into immune-deficient mice and then maintained by passaging from mouse to mouse. These models retain accurately the genetic, histological, and molecular characteristics of the original tumor and their response to treatments is correlated with clinical response [20]. However, they have some limitations, such as a low success rate of establishment for some tumor types, the long time required for the establishment, the time-consuming and costly process of their use, as well as the ethical issues associated to animal experimentation [21]. They offer therefore a suitable tumor model for testing innovative therapies but the above-mentioned limitations could restrict the use of these models for predictive purposes. However, their predictive value is currently tested in some clinical trials, as well as ex vivo models [12].

Among the ex vivo approaches, the technique of explants (or tumor slices) derives from the originally described technique of floating brain sections [22]. This model is obtained by cutting fresh tumor samples into slices 250 to 350 μm thick using a vibratome, and cultured ex vivo at 37 °C. The use of tumor slices maintains tumor-stroma interactions while preserving a tissue architecture that mimics the reality of the tumor in the short term. Despite a lack of reproducibility due to tumor heterogeneity, a study demonstrated the value of this model for predicting patient’s response to different anticancer agents [17] or for identifying predictive signature [16].

Ascites-derived spheroids (ADS) could offer as well a promising cancer model to guide clinical decision making. Ascites is most frequently associated with ovarian, pancreatic, colorectal, liver cancers, and provides a unique opportunity to easily sample tumor cells from these cancer patients. In the ascites, tumor cells shed from the primary tumor or visceral and parietal peritoneal carcinosis, forming free-floating spheroids [23]. These spheroids are poorly described and their predictive value has not been investigated so far. These samples can be used to perform ex vivo assays to assess their sensitivity to treatments [24] and therefore represent a particularly interesting alternative to explants, since the cells are abundant and can be collected at various time during the therapeutic management.

Finally, patient-derived tumor organoids (PDTO) have emerged more recently, as preclinical models that have the potential to predict an individual patient’s response to treatment. They are developed from patient tumor cells following embedding in basement membrane matrix and cultured in a medium supplemented with a cocktail of growth factors and inhibitors of signaling pathways to recapitulate in vivo niche conditions and allow long term growth [14]. These models are able to closely reproduce the genetic and morphological heterogeneous composition of the cancer cells in the original tumor. They can be rapidly grown from small amount of tumor cells, such as needle biopsy, with a high success rate compared to other models [14]. More importantly, despite the lack of stromal cells, there are more and more evidence that PDTO can recapitulate clinical response of patients [25, 26], including ovarian cancer patients, although most of the studies were based on small sample size.

Therefore, it is crucial to develop relevant patient-derived tumor models (PDX, PDTO, explants and ADS) to evaluate new therapeutic strategies, identify predictive molecular signatures and to determine predictive value of ex vivo models in clinical studies based on larger patient cohorts. In this regard, our study will evaluate the feasibility of establishing these models and performing functional assay for drug testing and to compare their response to treatments to the clinical response of ovarian cancer patients.

## Method/Design

**Fig. 1:**
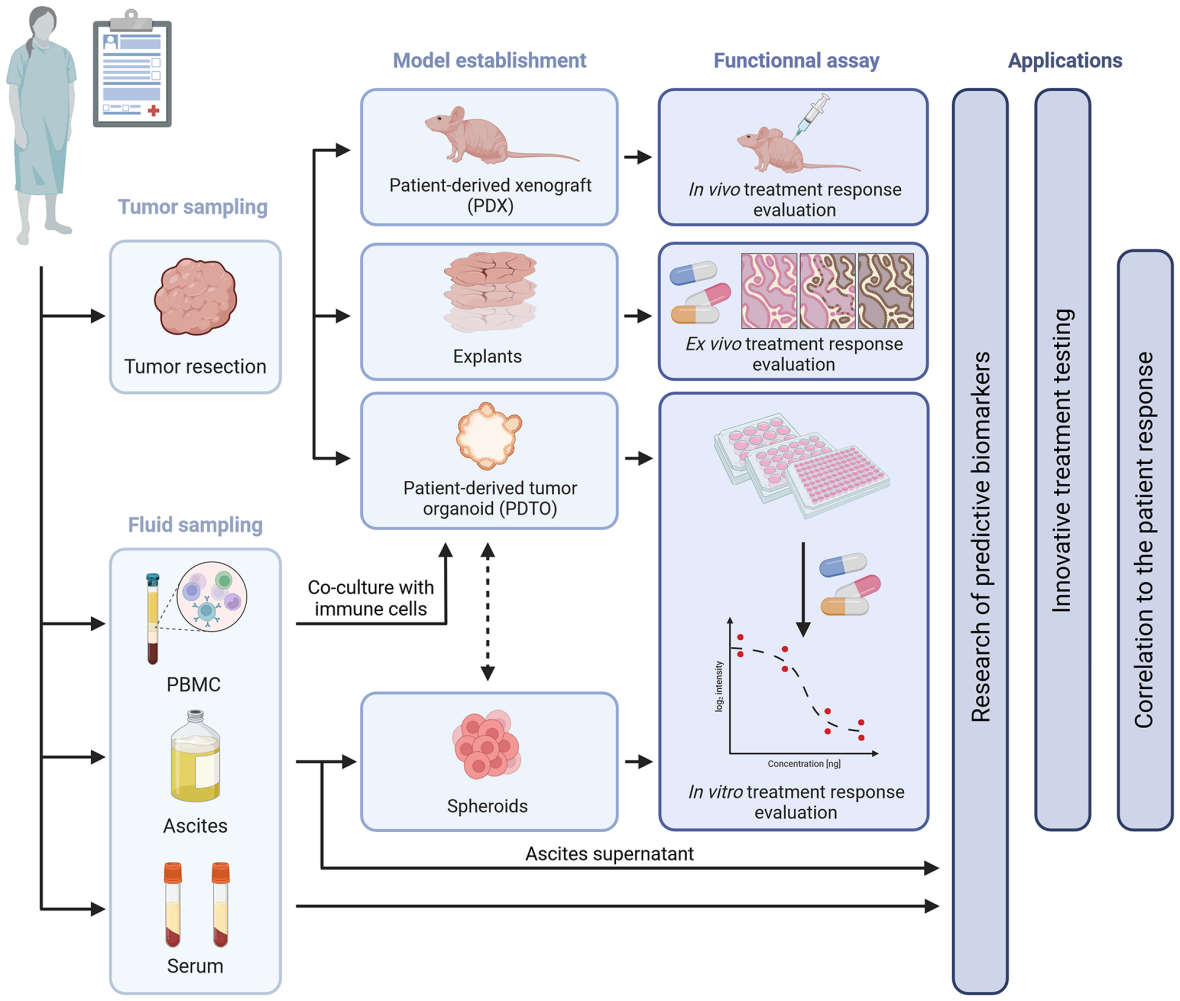
OVAREX study design (created with Biorender.com)

The OVAREX study is a single-center non-interventional study conducted at Comprehensive Cancer Centre François Baclesse (Caen, France) to investigate the feasibility of establishing and testing ex-vivo tumor models from ovarian cancer to predict clinical response of the patient (Fig. 1).

### Study objectives and endpoints

The main objective of the study is to assess the feasibility of developing ex-vivo tumor models that can be used for functional predictive assays.

The secondary objectives are to: *(i)* evaluate the effectiveness of ex vivo functional assays to predict the response to treatment; *(ii)* identify predictive biomarkers in tumor and serum samples; *(iii)* compare the ex vivo response of tumor models to clinical response; *(iv)* establish PDX models from ovarian cancer samples; *(v)* develop co-cultures of PDTO with autologous immune cells allowing the evaluation of anticancer effects of immunotherapy.

### Study population

Eligibility criteria are described in Table 1. The OVAREX study focuses on patients with proven cancer of the ovary, fallopian tube, and peritoneum, all FIGO stages (I-IV) who undergo laparoscopic or laparotomy surgery at our institution.

**Table 1:** OVAREX study inclusion and exclusion criteria

Inclusion criteria	Non-inclusion criteria
Patient over 18 years of age	Pregnant women
Patient with proven cancer of the ovary, fallopian tube or peritoneum	Persons deprived of liberty or under guardianship (including curatorship)
FIGO stages I to IV	History of any other clinically active malignancy in the last 5 years prior to inclusion
Laparoscopic or laparotomy surgery	
Patient affiliated to a social security system	
Patient informed and who expressed their non-opposition	

### Study assessment

The study was approved by the “East III” ethical committee (IDRCB: 2018-A02152-53). Clinicians will inform all patients enrolled in the study that their biological samples could be used for this study (specific information letter will be given to patients) and they will express their non-opposition. Moreover, we will obtain written informed consent from patients for the use of their biological samples for research purposes.

### Medical data collection

In order to correlate the biological data obtained on the initial tumor with the response to ex vivo treatments and the response observed in the clinic, the patients’ clinical data will be routinely collected from medical records by the Calvados Cancer Registry, which also checks for data completeness and consistency, and will be transmitted for enrolled patients. The collected data are summarized in Table 2.

**Table 2:** Medical data collected in the OVAREX study

Age
History of the disease (diagnosis, mutations status, management)
History of other cancer or not
Surgical procedure
Response to cancer treatments
Recurrence (type, date, location)
Date of death

This collection will be carried out from an already existing database which has been the subject of a prior declaration to the establishment’s French data protection authority (CNIL) representative. Indeed, a collection of samples annotated in terms of clinico-pathological parameters has been set up at the Centre François Baclesse in order to allow a correlation between the profile sensitivity to chemotherapy (conventional or innovative) and the parameters studied. The OVAREX project will therefore use pseudonymized data collected by our biological resource center for studies correlation between results obtained ex vivo and clinical data. The samples and associated data will be retrospectively collected at the Centre François Baclesse and stored in the Biological Resource Center (BRC) OvaRessources (NF-S 96,900 quality management, AFNOR No. 2016: 72860.5). All biological collections are declared to the MESR (Ministry of Education, Health and Research, France, No. DC-2010-1243).

### Collection of tumor and blood samples

#### Tumor

A laparoscopic surgery will be performed as part of the patient’s care and tumor sample will be collected for anatomopathological diagnosis. Tumor sample which is excess to diagnostic purposes will be sent directly to the laboratory in sterile vials filled with cold culture medium supplemented with a Rho-kinase inhibitor (Y-27632).

#### Ascites

As ascites can also be punctured during the surgery or outpatient hospitalization, excess fluid unneeded for anatomopathological evaluation will be collected in sterile jars and transferred to the laboratory.

#### Blood

Blood sampling will be realized before surgical intervention as part of the blood test included in the patient’s care. No blood draw will be done specifically for this study. Two dry tubes of 5 mL and 7 EDTA tubes of 5 mL will be collected and processed at the laboratory for serum analysis and peripheral blood mononuclear cells (PBMC) isolation.

### Biological sample processing

#### Tumor sample processing

Different procedures will be carried out on tumor samples: for future characterization, two pieces will be snap frozen and stored at -80 °C for molecular analyses and one piece will be fixed in paraformaldehyde for paraffin embedding and subsequent histopathological analysis and immunohistochemistry. The rest of the tumor will be processed to establish different models as described hereafter. All tumor samples will be stored in the BRC ‘OvaRessources’. Histology of all samples will be confirmed by a certified pathologist.

#### Isolation of PBMC

PBMC will be isolated from blood by density gradient centrifugation using Ficoll-Paque in Leucosep tubes. Cells will be resuspended in cold culture media, and counted. PBMC will be then resuspended in freezing solution (10% DMSO, 90% FBS), aliquoted (about 5 cryovials, 4.10^6^ cells/cryovial), and frozen with gradually decreasing temperatures (1 °C/min) to -80 °C before long-term storage at liquid nitrogen temperatures and stored in the BRC TCBN.

### Establishment and culture of PDTO, PDX, explants and ADS

#### PDTO establishment

Tumor samples and ascites will be processed as previously described [27]. Briefly, samples are mechanically and/or enzymatically dissociated to obtain single cells or small cell clusters. Cells will then be embedded in extracellular matrix BME2 and cultured in an enriched medium [Advanced DMEM (Gibco) supplemented with 100 UI/mL of penicillin and streptomycin (Gibco), 1% GlutaMAX (Gibco), 1X B27 (Gibco), 10 mM Nicotinamide (Sigma-Aldrich), 1.25 mM N-Acetyl-L-Cysteine (Sigma-Aldrich), 50 µg/mL Primocin (InvivoGen), 5 µM Y27632 (Interchim), 20 ng/mL FGF-10 (PeproTech), 500 nM A-83–01 (PeproTech), 50 ng/mL EGF (PeproTech), 1 ng/ml FGF-basic (PeproTech), 1 µM SB202190 (PeproTech), 1 µM PGE2 (Sigma-Aldrich), 10% RSPO1- conditioned media (Cultrex HA-R-Spondin1-Fc 293 T, Amsbio) and 50% L-WRN- conditioned media (Cultrex L-WRN, Amsbio)]. Culture medium will be changed every 3–4 days and PDTO passaged every 2–4 week in order to expand them. PDTO lines will be considered as established when they will be cultured for more than 3 passages. For each established PDTO line, samples will be kept frozen for DNA/RNA/protein analysis, others will be embedded in paraffin for histopathological analysis and dissociated cells will be biobanked at -150 °C.

#### PDX establishment

Immediately following patient’s surgery, tumor fragments will be subcutaneously engrafted into the scapular area of anaesthetized nude mice as previously described [26]. Tumor growth will be measured twice a week and serial fragment grafts of each tumor will be conducted on 3 to 5 athymic nude mice. When the tumors reach a volume of 800 to 1000 mm^3^, tumors will be harvested, one fragment will be fixed for paraffin embedding and histopathological/immunochemistry analyses, two pieces will be snap frozen and stored at -150 °C for DNA/RNA extractions and three pieces will be used for passage, residual fragments will be frozen in 10% (v/v) dimethylsulfoxid (DMSO) and 90% (v/v) fetal bovine serum (FBS).

#### Explants

As described by Lheureux et al. [16], vibratome-sliced nodes (300–400 μm) will be fixed with 3% paraformaldehyde, frozen at -80 °C for immunoblotting or transferred into sterile prewarmed complete culture medium (RPMI 1640 supplemented with 2 mM GlutamaxTM, 25 mM HEPES, 10% fetal calf serum, 33 mM sodium bicarbonate (Fisher Scientific Bioblock, Illkirch, France) and 1% antibiotic).

#### ADS culture

Following patient paracentesis, ascites will be centrifugated at 1300 g for 7 min, the supernatant will then be filtered using a 300 μm and a 50 μm sieves to retrieve the spheroids contained in ascites. Spheroids will be fixed in 3% PFA, frozen at -80 °C, biobanked at -150 °C or cultured in agarose-coated plate with the ascites supernatant obtained after filtration.

#### Coculture of PDTO with immune cells

PDTO specific autologous T cells will be induced according to modified version of the protocol described in Dijkstra et al. [28]. Briefly, PBMC will be activated with the corresponding PDTO lysate and specific T cells clones will be isolated based on their expression of CD154 and CD137 markers using flow cytometry sorting. Once isolated and their purity controlled, specific T cells will be amplified by the use of a stimulation matrix and then cryopreserved. A quality control will be performed before cryopreservation by flow cytometry to check for reactivity against PDTO using CD107a expression and cytokines production after antigen re-stimulation. Once produced and checked for antigen specificity, PDTO-specific T cells will be cocultured with PDTO to produce iPDTO for the evaluation of response to immunotherapy.

### Evaluation of the response of tumor-derived models to treatment

#### PDTO treatment

When PDTO reached the size of 75–150 μm in diameter, they will be collected and resuspended in PDTO treatment medium (PDTO culture medium lacking primocin, Y-27,632 and N-acetylcysteine) with 2% BME2. 200 PDTO per well will be seeded in 100 µL volume in a previously coated (1:1 PDTO treatment medium/BME2) white clear bottom 96-well plates (Greiner). Drug solutions will then be prepared in a 2% BME2/PDTO treatment medium, added to each well and plates will be transferred to a humidified 37 °C/5% CO2 incubator. During the treatment, PDTO will be monitored using IncuCyte S3 ZOOM (Sartorius). One week later, ATP levels will be measured by CellTiter-Glo 3D assay (Promega) and luminescence will be quantified using GloMax Discover Microplate Reader (Promega). The half-maximal inhibitory concentration (IC50) and the area under the dose-response curve (AUC) will be computed for each PDTO model.

#### PDX treatment

PDX fragments will be subcutaneously implanted into nude mice as described above. On the first day of treatment, the animals bearing 100 to 200 mm^3^ tumors will be randomly distributed to the various treatment and control groups (8–10 mice per group). Drugs will be administered intraperitoneally. Mice will be weighed and tumor volumes will be determined once or twice weekly from two-dimensional caliper measurements using the equation: Tumor volume (mm^3^) = [length (mm) x width (mm)^2^]/2. After 28 days of treatment, the mice will be euthanized and the tumors will be harvested for analysis. These experiments will be performed under guidelines from the European Community Council (2010/63/EU) and are approved by the protocol APAFIS #9577 validated by the French ethics committee “Comité d’éthique de Normandie en matière d’expérimentation animale” (CENOMEXA).

#### Explants treatment

After the transfer into sterile prewarmed complete culture medium, slices will be treated in complete medium for 6 to 48 h in a 5% CO2 humidified atmosphere at 37 °C. Slices will then be fixed in PFA 3% and paraffin-embedded for further analyses including the immunohistochemical detection of cleaved caspase-3 in order to quantify apoptosis.

#### ADS treatment

Directly after filtration and spheroids seeding in agarose-coated plates, the spheroid will be exposed to treatments for 6 to 96 h. As for PDTO, ADS will be monitored using IncuCyte S3 ZOOM (Sartorius) and viability will be assessed using CellTiter-Glo 3D assay (Promega).

### Evaluation of PDTO model relevance and identification of potential predictive biomarkers

#### Transcriptomic analysis

RNA analysis will be performed according to the protocol described in Perréard et al. [29]. Briefly, total RNA will be extracted using the Nucleospin RNA kit (Macherey Nagel, Hoerdt) and libraries will be made with the QuantSeq 3’RNA Library Kit. Once produced, the final library will be purified and deposed on High sensitivity DNA chip to be controlled on Agilent bioanalyzer 2100 and sequenced on NovaSeq 6000 (Illumina). Elimination of poor-quality regions and poly(A) of reads will be done through the use of the fastp program. Read alignments will be performed using the program STAR with the human genome reference (GRCh38) and the Ensembl reference gene annotations. Reads counts will be obtained using FeatureCount and statistical analysis will be realized with the R/bioconductor package DESeq2.

#### Copy number variation (CNV) analysis by low-pass whole genome sequencing (WGS)

WGS will be performed using Illumina DNA PCR Free prep kit, starting with 500ng of DNA. Data will be analyzed with HMMcopy and ichorCNA.

#### Transcriptome and CNV analysis

Analysis of intra reproducibility and differences between original tumors or ascites and PDTO will be assessed by principal component analysis and unsupervised hierarchical clustering as described in Perréard et al. [29].

#### Panel BRCAness

In order to assess tumors’ homologous recombination (HR) status, tumors will be sequenced with a 127-genes panel including 15 h genes (*BRCA1, BRCA2, ATM, BARD1, BRIP1, CDK12, CHEK1, CHEK2, FANCL, PALB2, PPP2R2A, RAD51B, RAD51C, RAD51D, RAD54L*). The sequencing data will be also used to determine a genomic instability score (GIS) as described by Leman et al. [10].

### Statistical consideration

#### Sample size determination

To estimate the PDTO establishment rate, assumed around 90%, with a 95% confidence interval of 10% width, 141 tumor samples will be required. Anticipating non-assessable samples, it is planned to include 250 patients.

#### Statistical analyses

Qualitative variables will be described using the sample numbers and percentages. Quantitative variables will be described using the mean (+/- standard deviation) or the median and the range if normality hypothesis is not verified. The significative threshold is set to 5% for all statistical analysis and confidence interval.

To address the primary objective, the rate of successful PDTO establishment, i.e., the rate of tumor samples usable for predictive functional assays based on PDTO, will be estimated with its 95% confidence interval. Then, association between PDTO response to treatment and clinical response will be measured by the Chi2 test. Associations between biological parameters and clinical response will be assessed by one-way analysis of variance (or the non-parametric Kruskal-Wallis test, if necessary). Receiver Operating Characteristic (ROC) curves and a logistic regression model will also be used to identify predictive factors of clinical response. Survival curves will be estimated by using the Kaplan-Meier method; median survival and survival rates at different times will be provided with their confidence intervals.

## Discussion

Current approaches to precision oncology are mainly based on the detection of genomic alterations. Unfortunately, many patients still do not benefit from these approaches despite the presence of an actionable alteration [12]. The use of personalized tumor models, such as PDX, PDTO, spheroids or explants, is rapidly emerging as a strategy to complement the use of genomics. These models could be used for drug testing as part of a predictive functional assay to guide clinical decision making, as well as to test innovative therapeutic strategies and identify predictive biomarkers. Interestingly, responses of some ex vivo models to treatments have been positively correlated to patient responses [17, 25], including ovarian cancer patients [30]. However, these studies were based on small sample size and it is therefore crucial to determine if the response of these different personalized models to treatments recapitulate clinical response on large patient cohorts.

In this clinical study, we propose to establish PDX and ex vivo models (PDTO, ascites-derived spheroids, and explants) from tumors or ascites of ovarian cancer patients who will undergo surgical procedure or paracentesis. We aim at demonstrating the predictive value of the ex vivo models for their potential use in routine clinical practice as part of precision medicine. In the meantime, we want to establish tumor model collection of PDTO and PDX for new therapeutic compounds/strategies testing as well as for the identification of predictive biomarkers. Special attention will be given to immunotherapy testing using co-culture of PDTO with immune cells. This study will allow the establishment of a collection of relevant ovarian cancer models of various histology including rare ovarian cancer types and could demonstrate the interest of ex vivo models to predict the response to treatments or to identify innovative therapeutic strategies. In the event that one (or several) model(s) could faithfully predict patient response in a clinical-adapted manner (high success rate of establishment, results available within clinically relevant time frames, etc.), a prospective randomized clinical trial could be designed. The implementation of such predictive functional assay could thus allow individualizing cancer care and enabling physicians to select the most effective treatment for their patients.

## Data Availability

No datasets were generated or analysed during the current study.

## Abbreviations

ADS: Ascites-Derived Spheroids
AUC: Area Under the Curve
BME: Basement Membrane Extract
BRC: Biological Resource Center
BRCA: Breast Cancer
CNV: Copy Number Variations
DMEM: Dulbecco’s Modifed Eagle Medium
DNA: DeoxyriboNucleic Acid
EDTA: EthyleneDiamineTetraacetic Acid
EGF: Epidermal Growth Factor
EOC: Epithelial Ovarian Cancer
FBS: Fetal Bovine Serum
FGF: Fibroblast Growth Factor
FIGO: Federation of Gynecology and Obstetrics
HRD: Homologous Recombination Deficiency
IC50: Half maximal inhibitory concentration
IHC: ImmunoHisto Chemistry
NGS: Next-Generation Sequencing
PARPi: Poly (ADP-Ribose) Polymerase Inhibitor
PBMC: Peripheral Blood Mononuclear Cells
PDTO: Patient-Derived Tumor Organoid
PDX: Patient-Derived Xenograft
PFA: ParaFormAldehyde
RNA: RiboNucleic Acid
ROC: Receiver Operating Characteristic
WGS: Whole Genome Sequencing
